# Structure Based Refinement of a Humanized Monoclonal Antibody That Targets Tumor Antigen Disialoganglioside GD2

**DOI:** 10.3389/fimmu.2014.00372

**Published:** 2014-08-14

**Authors:** Mahiuddin Ahmed, Jian Hu, Nai-Kong V. Cheung

**Affiliations:** ^1^Department of Pediatrics, Memorial Sloan Kettering Cancer Center, New York, NY, USA

**Keywords:** antibody engineering, ganglioside, neuroblastoma, melanoma, structure, computational chemistry

## Abstract

Disialoganglioside GD2 is an important target on several pediatric and adult cancer types including neuroblastoma, retinoblastoma, melanoma, small-cell lung cancer, brain tumors, sarcomas, and cancer stem cells. We have utilized structural and computational methods to refine the framework of humanized monoclonal antibody 3F8, the highest affinity anti-GD2 antibody in clinical development. Two constructs (V3 and V5) were designed to enhance stability and minimize potential immunogenicity. Construct V3 contained 12 point mutations and had higher thermal stability and comparable affinity and *in vitro* tumor cells killing as the parental hu3F8. Construct V5 had nine point mutations to minimize potential immunogenicity, but resulted in weaker thermal stability, weaker antigen binding, and reduced tumor killing potency. When construct V3 was combined with the single point mutation HC:G54I, the resulting V3-Ile construct had enhanced stability, antigen binding, and a nearly sixfold increase in tumor cell killing. The resulting product is a lead candidate for clinical development for the treatment of GD2-positive tumors.

## Introduction

GD2 is a ganglioside expressed in several pediatric and adult cancer types and has been actively targeted by cancer immunotherapy approaches (see Ref. (1) for recent review). GD2 is a member of the b-series gangliosides, which are normally expressed during fetal development and are highly restricted to the central nervous system in healthy adults, with low levels of expression on peripheral nerves and skin melanocytes (2). GD2 has been found to be expressed in neuroectoderm-derived tumors and sarcomas, including neuroblastoma, retinoblastoma, melanoma, small-cell lung cancer, brain tumors, and sarcomas (3–5). Recent evidence has also shown that GD2 can be found on breast cancer stem cells (6, 7), as well as on neuroectodermal (8) and mesenchymal stem cells (9, 10).

Because of its surface expression on tumor cells and restricted normal expression in the brain and low levels in the periphery, GD2 has been an ideal target for the development of monoclonal antibodies (MoAbs), which cannot cross the blood–brain barrier. Several anti-GD2 antibodies have been developed and tested in the clinic over the past 20 years, primarily in pediatric neuroblastoma patients. 3F8 was the first anti-GD2 MoAb to be tested in patients with neuroblastoma (3, 11, 12). MoAb 3F8 is a murine IgG3 with the highest reported affinity for GD2 (*K*_D_ = 5 nM) (13). It binds specifically to the pentasaccharide epitope on GD2. Phase II clinical data have demonstrated that 3F8 when combined with the cytokine GM-CSF can significantly improve the survival of high-risk stage 4 children with metastatic neuroblastoma (14). Murine 3F8 was more recently humanized (hu3F8) based on complementarity determining region (CDR) grafting (13), and is currently in Phase I clinical trials (clinical trials.gov NCT01419834, NCT01757626, and NCT01662804).

We have previously solved the crystal structure of murine 3F8 to 1.65 Å resolution (protein data bank 3VFG) and used completely *in silico* methods to find a single point mutation (HC:G54I) that could significantly enhance the antibody-dependent cell-mediated cytotoxicity (ADCC) of hu3F8 (15). Based on computational modeling, we have developed two additional hu3F8 frameworks, named V3 and V5, which were designed to optimize the properties of hu3F8. More specifically, V3 was designed to maximize stability and V5 was designed to minimize potential immunogenicity. We present here the computational methods used to derive the hu3F8 V3 and V5 frameworks along with their experimental properties of antigen binding, thermal stability, and *in vitro* ADCC.

## Materials and Methods

### Molecular modeling

Molecular modeling, energy calculations, and image renderings were done using Discovery Studio 4.0 (Accelrys, San Diego, CA, USA). The crystal structure of m3F8 Fab (pdb 3VFG) and the homology model of hu3F8 Fab were simulated using CHARMm (CHemistry at Harvard Molecular mechanics) force fields, and the effects of point mutations were calculated from the difference between the folding free energies of the mutated structure and the parental protein. Generalized Born approximation was used to account for the effect of the solvent and all electrostatic terms were calculated as a sum of coulombic interactions and polar contributions to the solvation energy. A weighted sum of the van der Waals, electrostatic, entropy, and non-polar terms was calculated for each point mutation.

### Construction and expression of hu3F8 constructs

Humanized 3F8 genes were synthesized for CHO cells (Blue Heron Biotechnology or Genscript) as previously described (13). Using the bluescript vector, these heavy and light chain genes of hu3F8 were transfected into DG44 cells and selected with G418 (InVitrogen, CA, USA). Hu3F8 producer lines were cultured in Opticho serum free medium (InVitrogen) and the mature supernatant was harvested as previously described (13). Protein A affinity column was pre-equilibrated with 25 mM sodium citrate buffer with 0.15 M NaCl, pH 8.2. Bound hu3F8 was eluted with 0.1 M citric acid/sodium citrate buffer, pH 3.9 and alkalinized (1:10 v/v ratio) in 25 mM sodium citrate, pH 8.5. It was passed through a Sartobind-Q membrane and concentrated to 5–10 mg/mL in 25 mM sodium citrate, 0.15 M NaCl, pH 8.2.

### Thermal stability measurements

The thermal stabilities of MoAbs were measured by differential scanning fluorimetry using the Protein Thermal Shift assay (Life Technologies). MoAbs (0.2 mg/mL) were mixed with Sypro Orange dye and fluorescence was monitored using a StepOnePlus quantitative PCR machine (Applied Biosystems) with a 1% thermal gradient from 25 to 99°C. Data were analyzed using Protein Thermal Shift Software (Applied Biosystems) to calculate the Tm using the derivative method. Fab and F(ab’)2 preparations of hu3F8 were used to correctly assign the Fab peak for the hu3F8 samples. All samples were prepared in triplicate. Statistical significance was calculated using a student’s *T* test.

### Binding kinetics by surface plasmon resonance

*In vitro* binding kinetics were measured using Biacore T-100 (GE Healthcare) as previously described (13). In brief, gangliosides were directly immobilized onto the CM5 sensor chip via hydrophobic interaction. Purified anti-GD2 MoAbs were diluted in HBS-E buffer containing 250 mM NaCl at increasing concentrations (50–1600 nM) prior to analysis. Samples (60 μL) were injected over the sensor surface at a flow rate of 30 μL/min over 2 min. Following completion of the association phase, dissociation was monitored in HBS-E buffer containing 250 mM NaCl for 300 s at the same flow rate. At the end of each cycle, the surface was regenerated using 50 μL 20 mM NaOH at a flow rate of 50 μL/min over 1 min and 100 μL 4 M MgCl_2_ at a flow rate of 50 μL/min over 2 min. The data were analyzed by the bivalent analyte model and default parameter setting for the rate constants using the Biacore T-100 evaluation software, and the apparent association on rate constant (*k*_on_), dissociation off rate constant (*k*_off_), and equilibrium dissociation constant (*K*_D_ = *k*_off_/*k*_on_) were calculated.

### Antibody-dependent cell-mediated cytotoxicity by ^51^chromium release

Human neuroblastoma cell line LAN-1 was provided by Dr. Robert Seeger (Children’s Hospital of Los Angeles). LAN-1 cells were grown in F10 RPMI 1640 medium supplemented with 10% fetal bovine serum (Hyclone, South Logan, UT, USA), 2 mM glutamine, 100 U/mL penicillin, and 100 μg/mL streptomycin at 37°C in a 5% CO_2_ incubator. ADCC assays were performed using NK-92MI cells stably transfected with the human CD16 Fc receptor as previously described (13). LAN-1 target cells were detached with 2 mM EDTA in Ca^2+^ Mg^2+^ free PBS and washed in F10, before radiolabeling with ^51^Cr for ADCC assays. All samples were prepared in triplicate. Dose–response curves were fitted by non-linear regression to a sigmoidal dose–response (variable slope) model, using GraphPad Prism software, to allow for determination of EC50. For comparison of curves, best-fit values for EC50 were analyzed for significance using F tests.

## Results

### Design of constructs V3 and V5

**Figure 1:**
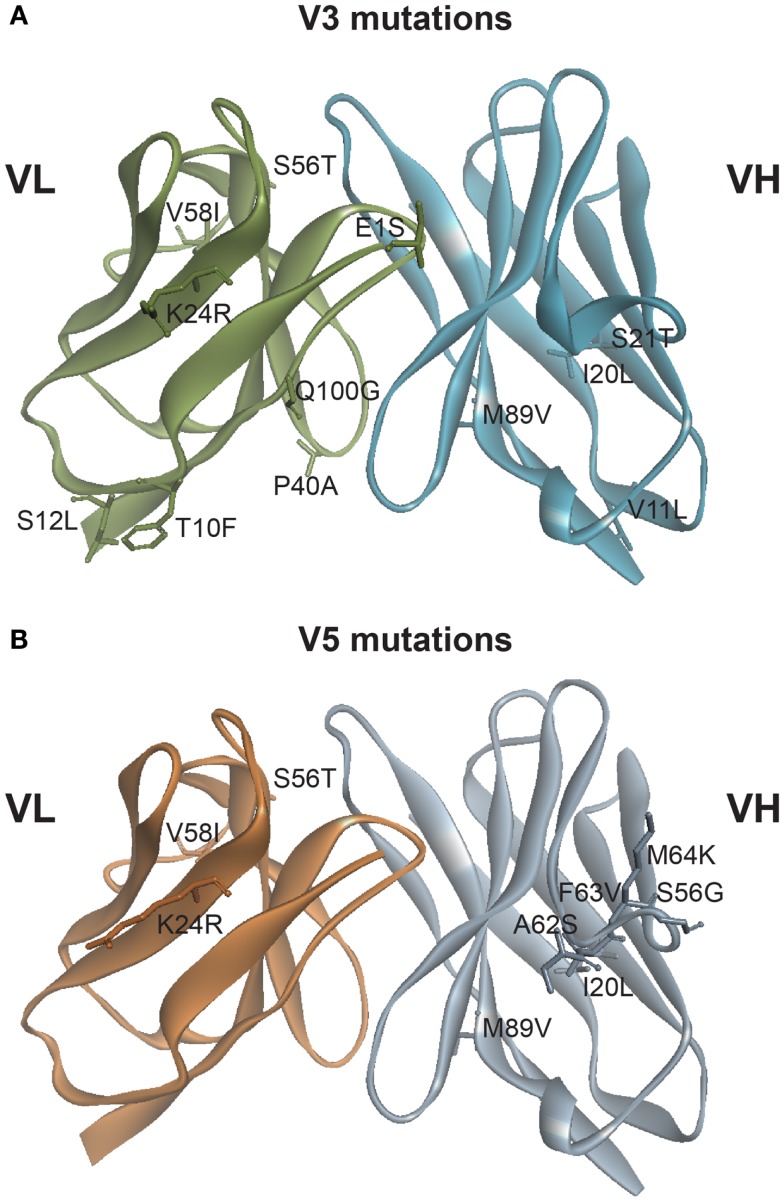
**Mutations generated based on *in silico* modeling**. **(A)** Location of 12 point mutations in hu3F8 for construct V3. **(B)** Location of nine point mutations in hu3F8 for construct V5. Full listing of mutational energies can be found in Tables 1 and 2.

Constructs V3 and V5 (see Figure 1) were designed utilizing completely *in silico* methods, based on both the crystal structure of murine 3F8 Fab (pdb 3VFG) and a homology model of hu3F8 Fab that was built using MODELLER followed by CHARMm energy minimizations. The original hu3F8 that was built by CDR grafting methods utilized the human germline sequences IGHV3-33 for the heavy chain template and IGKV3-15 for the light chain template (www.imgt.org). These same templates were utilized in deciding which mutations to incorporate into V3 and V5, in order to minimize potentially immunogenic sequences.

**Table 1:** **Mutation energies associated with the design of construct V3**.

Mutation	Location	Mutation energy (kcal/mol) in m3F8 Fab	Mutation energy (kcal/mol) in hu3F8 Fab	Resulting phenotype
LC: E1S	Framework	−0.48	+0.28	Murine
LC: T10F	Framework	+0.17	−0.95	Murine
LC: S12L	Framework	−1.26	+0.79	Murine
LC: K24R	CDR L1	−0.21	−0.17	Human
LC: P40A	Framework	+0.22	+1.41	Murine
LC: S56T	CDR L2	−0.05	+0.04	Human
LC: V58I	Framework	−0.64	+0.01	Human
LC: Q100G	Framework	+1.06	+2.94	Murine
HC: V11L	Framework	−1.61	+0.99	Murine
HC: I20L	Framework	+0.43	−0.86	Human
HC: S21T	Framework	−0.68	+0.42	Murine
HC: M89V	Framework	−0.50	−0.29	Human
**Net result**		***−*3.55**	*****+***4.61**	

**Table 2:** **Mutation energies associated with the design of construct V5**.

Mutation	Location	Mutation energy (kcal/mol) in m3F8 Fab	Mutation energy (kcal/mol) in hu3F8 Fab	Resulting phenotype
LC: K24R	CDR L1	−0.21	−0.17	Human
LC: S56T	CDR L2	−0.05	+0.04	Human
LC: V58I	Framework	−0.64	+0.01	Human
HC: I20L	Framework	+0.43	−0.86	Human
HC: A62S	CDR H2	−0.24	−0.50	Human
HC: F63V	CDR H2	+1.91	+1.80	Human
HC: M64K	CDR H2	+0.10	−0.23	Human
HC: S65G	CDR H2	+0.82	+1.10	Human
HC: M89V	Framework	−0.50	−0.29	Human
**Net result**		*****+***1.62**	*****+***0.90**	

Table 1 shows the 12 mutations that were incorporated into hu3F8 resulting in construct V3, along with their predicted mutational energies. *In silico* mutagenesis was done on every potential humanizing mutation in the murine 3F8 structure that was not directly predicted to be involved in antigen recognition, based on our previous 3F8:GD2 docked model (15). In addition, potential back mutations and humanizing mutations were analyzed in the homology model of hu3F8. Table 1 shows both of these sets of calculations. In choosing which mutations to incorporate into construct V3, more emphasis was placed in the first set of calculations for stabilizing the murine 3F8 structure, since this was the experimentally verified high-resolution crystal structure, in comparison to the homology model of hu3F8, which can contain inherent error. Another consideration in placing emphasis on the native structure of murine 3F8 was the fact that murine 3F8 had consistently shown higher antigen-binding affinity than hu3F8 (13).

The analysis showed that five mutations that were made in the original hu3F8 were destabilizing, and so for construct V3, those mutations were reverted back to the murine sequence (LC:E1S, LC:T10F, LC:S12L, HC:V11L, and HC:S21T). Two additional back mutations were made (LC:P40A and LC:Q100G) because they involved Gly or Pro residues that can affect protein backbone conformation. To offset the potential immunogenicity of these seven back mutations in the V3 construct, five humanizing mutations were added (LC:K24R, LC:S56T, LC:V58I, HC:I20L, and HC:M89V), which had either enhanced stability or had a negligible effect ( < 0.5 kcal/mol). Two of these mutations involved mutating CDR residues (LC:K24R and LC:S56T). The net result of all 12 mutations was predicted to have a stabilizing mutational energy of -3.55 kcal/mol to the murine 3F8 structure. However, this same set of mutations in the model of hu3F8 was predicted to have a destabilizing mutational energy of +4.61 kcal/mol.

Table 2 shows the nine point mutations were incorporated into hu3F8 to make construct V5, in an effort to minimize potential immunogenicity. In addition to the five humanizing mutations from construct V3 (LC:K24R, LC:S56T, LC:V58I, HC:I20L, and HC:M89V), construct V5 also includes four additional humanizing mutations (HC:A62S, HC:F63V, HC:M64K, and HC:S65G), which are located on CDR H2. These four CDR residues were predicted to be a part of a potentially moderate affinity MHC class II T-cell epitope, which can result in enhanced immunogenicity (as identified using the NN-align method on the Immune Epitope Database (http://www.iedb.org/). The net mutational energy of all nine mutations in construct V5 was predicted to be a moderately destabilizing +1.62 kcal/mol for the murine 3F8 structure, and +0.90 kcal/mol for the hu3F8 model.

Potential immunogenicity of constructs V3 and V5 as compared to hu3F8 was analyzed using the T20 score analyzer (16), a new *in silico* tool that can predict the “humanness” content of antibody variable regions derived from a database of ~38,700 human antibody variable sequences. Table 3 shows the T20 scores for hu3F8, V3, and V5. As expected, the net two additional murine mutations in V3 compared to hu3F8 resulted in slightly lower T20 scores, and the net nine humanizing mutations in V5 resulted in higher T20 scores, a characteristic of low immunogenicity MoAbs.

**Table 3:** **Humanness content based on T20 score analyzer (16)**.

Domain	Construct	T20 (CDR + framework)	T20 (framework)
VL	hu3F8	76.0	84.8
VL	V3	74.8	80.2
VL	V5	78.9	85.9
VH	hu3F8	71.6	82.2
VH	V3	71.6	82.0
VH	V5	76.4	84.3

*Scale is on the order of 0–100, with 100 being the most human in sequence*.

### Thermal stability

The thermal stability of hu3F8, V3, and V5 was measured using differential scanning fluorimetry (see Table 4). The Fab domain of construct V3, which was designed to be more stable, had a nearly 2°C increase in Tm compared to hu3F8 (*p* = 0.006). Construct V5, on the other hand, had substantially lower thermal stability (9°C lower Tm than hu3F8). Based on the enhanced stability, V3 was chosen as a lead candidate, and the HC:G54I mutation, which we had previously shown to enhance tumor cell killing, was incorporated to make construct V3-Ile. The measured thermal stability of V3-Ile was nearly identical to V3.

**Table 4:** **Thermal stability of hu3F8 constructs**.

Construct	Fab Tm (°C)	ΔTm Fab (°C)	*p-*Value
hu3F8	73.6 ± 0.3		
V3	75.4 ± 0.5	1.8	0.006
V3-Ile	75.1 ± 0.1	1.5	0.001
V5	64.5 ± 0.3	−9.1	< 0.001

*Samples were prepared in triplicate and measured by differential scanning flourimetry. Values are shown as mean ± standard deviation*.

### Antigen binding kinetics

**Figure 2:**
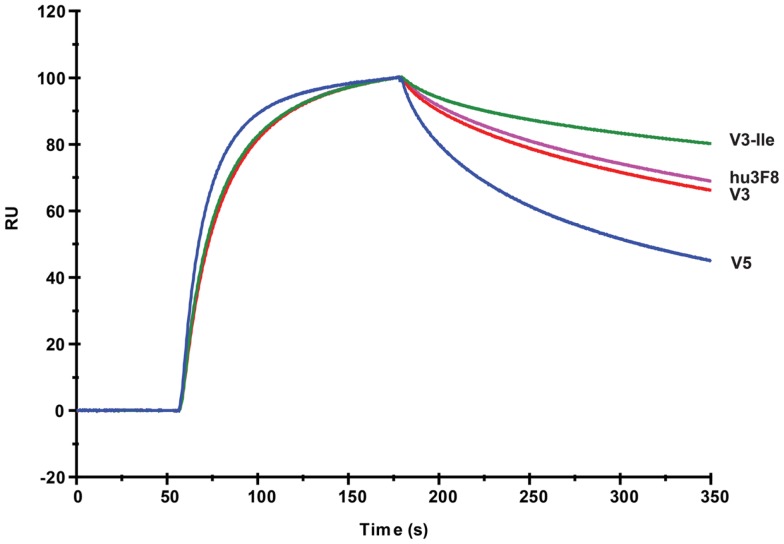
**Composite surface plasmon resonance sensorgram of the binding hu3F8, V3, V3-Ile, and V5 to ganglioside GD2**. Analysis of binding kinetics is shown in Table 5. Full sensorgrams are presented in Figure S1 in Supplementary Material.

The GD2 binding kinetics of hu3F8, V3, V3-Ile, and V5 were measured by surface plasmon resonance (see Figure 2 for normalized composite sensorgram, Figure S1 in Supplementary Material for complete sensorgrams, and Table 5 for analysis). Construct V3 (11.5 nM *K*_D_) had similar binding properties to hu3F8 (9.1 nM *K*_D_). Construct V5, on the other hand, had an almost twofold loss in binding (19.1 nM *K*_D_), which may have resulted from the additional CDR mutations and/or the weakened thermal stability. Construct V3-Ile had the highest GD2 affinity (3.7 nM *K*_D_). Interestingly, this enhancement in affinity is higher than what we had previously observed with the HC:G54I mutation in the parental hu3F8 framework.

**Table 5:** **Analysis of binding kinetics measured by surface plasmon resonance**.

Construct	*K*_on_ (S^-1^ M^-1^)	*K*_off_ (S^-1^)	*K*_D_ (nM)
hu3F8	1.15 × 10^5^	1.04 × 10**^-^**^3^	9.1
V3	1.09 × 10^5^	1.25 × 10**^-^**^3^	11.5
V3-Ile	1.28 × 10^5^	0.48 × 10**^-^**^3^	3.7
V5	1.73 × 10^5^	3.30 × 10**^-^**^3^	19.1

**K*_on_ and *K*_off_ were determined from individual sensorgrams, shown in Figure S1 in Supplementary Material*.

### *In vitro* antibody-dependent cell-mediated cytotoxicity

**Figure 3:**
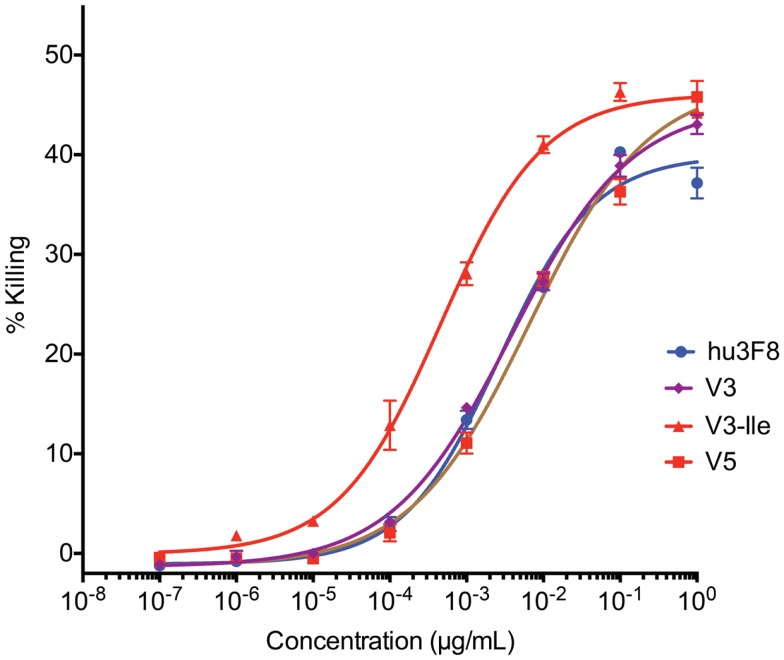
***In vitro* antibody-dependent cell-mediated cytotoxicity assay of human neuroblastoma LAN-1 target cells**. Antibodies were applied to LAN-1 cells in the presence of NK-92MI cells stably transfected with the human CD16 Fc receptor, at an effector:target ratio of 20:1. Samples were prepared in triplicate, and cytotoxicity was measured by ^51^chromium release. Values are shown as mean ± standard error.

Antibody-dependent cell-mediated cytotoxicity assays were done to test the effectiveness of hu3F8, V3, V3-Ile, and V5 on human neuroblastoma LAN-1 cells (see Figure 3 and Table 6). Cytotoxicity of an isotype matched non-targeting control is shown in Figure S2 in Supplementary Material. Consistent with the antigen binding data, V3 had similar binding to hu3F8 (EC_50_ of 3.83 ± 0.51 × 10^-3^ μg/mL for V3 compared to EC_50_ of 2.61 ± 0.48 × 10^-3^ μg/mL for hu3F8, *p* = 0.1138). Construct V5 had significantly weaker killing (EC_50_ of 6.55 ± 1.45 × 10^-3^ μg/mL, *p* = 0.0025). Construct V3-Ile had the highest level of killing (EC50 of 0.46 ± 0.08 × 10^-3^ μg/mL), a nearly sixfold increase in killing relative to hu3F8 (*p* < 0.0001) and eightfold increase over its parental V3.

**Table 6:** **Analysis of *in vitro* antibody-dependent cell-mediated cytotoxicity of neuroblastoma LAN-1 cells**.

Construct	EC50 (×10^-3^ μg/mL)	Relative potency	*p-*Value
hu3F8	2.61 ± 0.48	1	
V3	3.83 ± 0.51	0.7	0.1138
V3-Ile	0.46 ± 0.08	5.7	<0.0001
V5	6.55 ± 1.45	0.4	0.0025

*Samples were prepared in triplicate. Values are shown as mean ± standard error*.

## Discussion

Aberrant glycosylation has long been considered to be a hallmark of cancer (17). Ganglioside markers such as GD2 have become an attractive target in recent years because of the number of tumor types and cancer stem cells that express it on their surface, as well as GD2’s restricted expression in normal tissue. Monoclonal antibody 3F8 is a lead therapeutic candidate in this area, and its derivatives are being tested in a number of different targeting strategies including bispecific T-cell engaging antibodies, pre-targeted radio-immunotherapy, drug/toxin conjugates, nanoparticles, and even chimeric antigen receptors for use in adoptive cell therapy.

As in all antibody therapeutics, *in vivo* efficacy is affected by antigen affinity, antibody stability, immunogenicity, as well as a number of serum stability and pharmacokinetics related factors. In this study, we have investigated a structural and computational approach to refine the stability and to reduce computationally predicted immunogenicity of a humanized form of the anti-GD2 MoAb 3F8. By introducing site-specific mutations based on force-field simulations of the antibody crystal structure, we generated construct V3, which had significantly higher thermal stability, and comparable antigen binding and *in vitro* ADCC.

We have additionally attempted to minimize potential immunogenicity in designing construct V5, which had major mutations to CDR residues to eliminate a predicted T-cell epitope. Immunogenicity is a major component of clinical efficacy. A large percentage of neuroblastoma patients treated with the murine 3F8 developed a human anti-mouse antibody response, limiting repeated administrations of this antibody. In the case of designing construct V5, however, the mutations were too stringent, resulting in lowered thermal stability, weaker antigen binding, and weaker tumor cell killing. Finally, we combined our stability enhanced V3 construct with the cytotoxicity enhancing mutation (HC:G54I), which resulted in enhanced stability, antigen binding, and *in vitro* tumor cell killing, compared to the parental hu3F8.

While there are several examples of using computational methods to enhance the properties of antibodies [see Ref. (18) for review], there are few examples of using site-specific *in silico* based framework mutations to enhance thermal stability profiles. Wang and Duan (19) did suggest mutations to the VH–VL interface of anti-VEGF single-chain variable fragment (scFv) to enhance thermal stability based on molecular dynamics simulations, but with no experimental validation. We have recently shown that disulfide stabilization at the VH–VL interface of the anti-GD2 scFv 5F11 in the context of a GD2xCD3 tandem scFv bispecific antibody resulted in a 10°C increase in thermal stability and a nearly 150-fold increase in tumor killing potency (20). Enhancing thermal stability can also lead to less aggregation and less immunogenicity. Liu et al. (21) have shown that disulfide stabilization of an anti-CD22 antibody–toxin fusion protein resulted in enhanced thermal stability and less immunogenicity in mice. What is novel and less obvious in this investigation is that we used *in silico* predictions to make site-specific framework mutations which resulted in a nearly 2°C increase in thermal stability to the V3 framework, and when combined with a cytotoxicity enhancing mutation also derived by *in silico* methods, resulted in enhancement of both antigen binding affinity and tumor cell killing potency. In fact, we had previously shown that cytotoxicity enhancing Ile mutation (HC:G54I) had nearly the same binding to GD2 as the parental hu3F8 (15), but when the same mutation was inserted into the more stable V3 framework in this investigation, there was a greater than twofold enhancement to GD2 binding. We have therefore demonstrated that structural and computational methods can be used to refine MoAbs that bind to complex carbohydrate targets such as GD2, with further *in vivo* validation necessary to progress toward clinical development.

## Conflict of Interest Statement

Mahiuddin Ahmed and Nai-Kong V. Cheung were named as inventors in patents related to antibody 3F8 filed by Memorial Sloan Kettering Cancer Center. Jian Hu declares that the research was conducted in the absence of any commercial or financial relationships that could be construed as a potential conflict of interest.

## Supplementary Material

The Supplementary Material for this article can be found online at http://www.frontiersin.org/Journal/10.3389/fimmu.2014.00372/abstract
