# Supplementary material for: Structure Based Refinement of a Humanized Monoclonal Antibody That Targets Tumor Antigen Disialoganglioside GD2
Source: Front Immunol. 2014 Aug 14;5:372. doi: 10.3389/fimmu.2014.00372 (PMC4132262; doi:10.3389/fimmu.2014.00372)
Supplement: Supplementary file 1 [file Presentation_1.PDF]

Supplementary Material

Structure based refinement of a humanized monoclonal antibody that targets tumor antigen disialoganglioside GD2

Mahiuddin Ahmed PhD[1], Jian Hu PhD[1], and Nai-Kong V Cheung MD PhD[1*]

[1]Department of Pediatrics, Memorial Sloan Kettering Cancer Center, New York, NY 10065, USA.

*Corresponding Author:
Nai-Kong V. Cheung, MD PhD
Department of Pediatrics
Memorial Sloan-Kettering Cancer Center
1275 York Avenue
New York, NY 10065
cheungn@mskcc.org
Phone: 646-888-2313
Fax: 646-422-0452

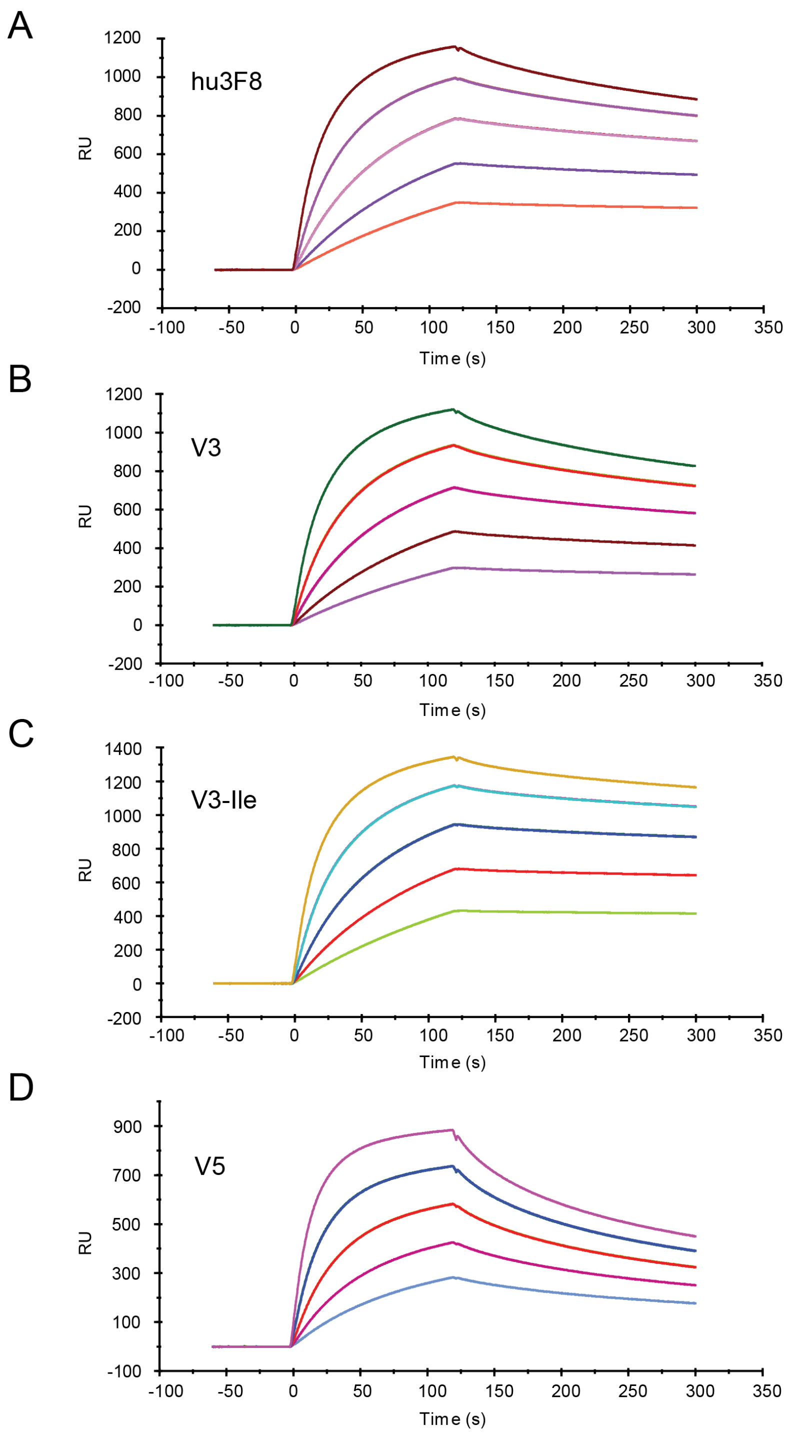

Figure S1. Surface Plasmon Resonance Sensorgrams. (A) hu3F8, (B) V3, (C) V3-Ile, and (D) V5. Sensorgrams are shown for antibody concentrations at 1600, 800, 400, 200, 100, and 50 nM for each of the samples.

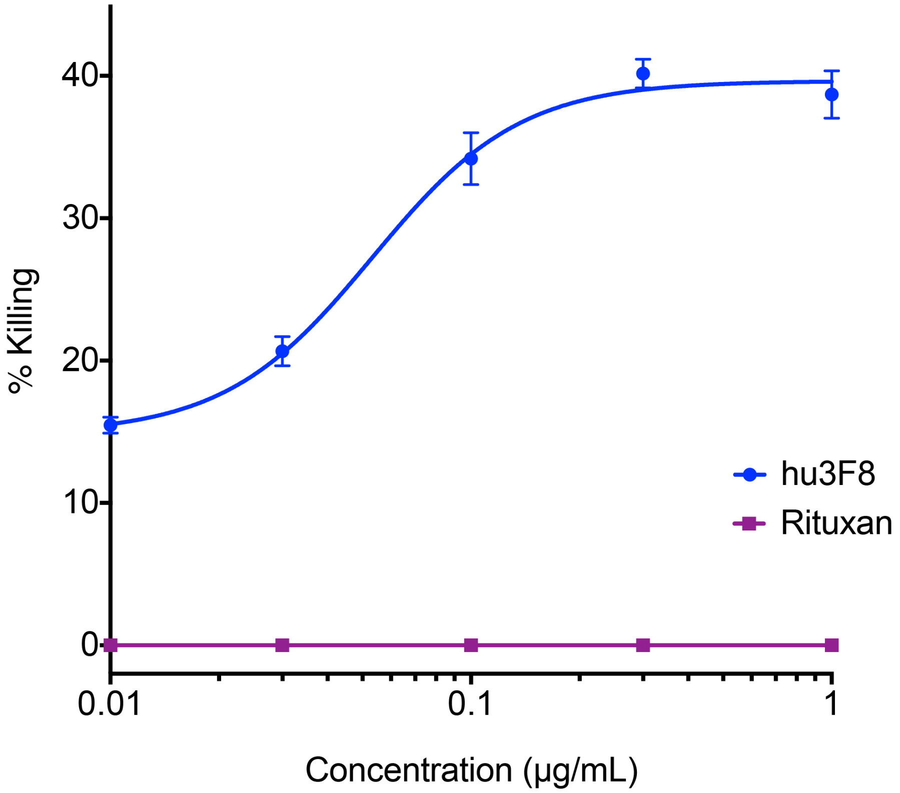

Figure S2. Control antibody-dependent cell-mediated cytotoxicity assay. Human neuroblastoma LAN-1 target cells were treated with hu3F8 or a non-targeting isotype matched control antibody, Rituxan (Rituximab), which is a chimeric MoAb with human IgG1 constant domains. Samples were prepared in triplicate and values are shown as mean ± standard error.
